# Granulomatosis With Polyangiitis Presenting With Kidney Failure: A Case Report

**DOI:** 10.7759/cureus.92380

**Published:** 2025-09-15

**Authors:** Konrad Haraziński, Weronika Goliat, Michalina Goliat-Mazurek, Dominik Mazurek, Izabela Jastrzebska, Anna Kaput, Kamil Poreba, Marcel Bobinski, Alicja Czyszczon, Aneta Tkaczyk

**Affiliations:** 1 Medicine, Medical University of Silesia in Katowice, Katowice, POL; 2 Medicine, Provincial Hospital in Poznań, Poznań, POL; 3 General Dentistry, Individual Medical and Dental Practice, Konary, POL; 4 Family Medicine, Kamionki Medical Center, Kamionki, POL; 5 Internal Medicine, Międzyleski Specialized Hospital in Warsaw, Warsaw, POL; 6 Internal Medicine, University Clinical Hospital in Opole, Opole, POL; 7 Internal Medicine, John Paul II Podhale Specialist Hospital in Nowy Targ, Nowy Targ, POL

**Keywords:** acute renal failure, gpa, granulomatosis with polyangiitis, hemodialysis, wegner’s granulomatosis

## Abstract

Granulomatosis with polyangiitis (GPA) is a rare and serious disease associated with antineutrophil cytoplasmic antibody (ANCA) antibodies. It can involve the kidneys and respiratory tract and may present as a pulmonary-renal syndrome. This report presents the case of a 57-year-old man admitted to the nephrology department with acute renal failure and systemic symptoms, including low-grade fever, malaise, and abdominal pain. During hospitalization, laboratory tests revealed rapidly progressive renal failure, elevated inflammatory markers, and oliguria. A subsequent kidney biopsy confirmed pauci-immune necrotizing glomerulonephritis, and serology was positive for PR3-ANCA, supporting the initial diagnosis of GPA. Immunosuppressive therapy was initiated and later adjusted due to complications. Despite these challenges, the patient’s clinical status improved, and he was discharged in stable condition.

## Introduction

Granulomatosis with polyangiitis (GPA), formerly known as Wegener's granulomatosis, is an autoimmune disease that results from antineutrophil cytoplasmic antibody (ANCA)-associated vasculitis. ANCA-associated vasculitis is a heterogeneous group of diseases that also includes eosinophilic granulomatosis with polyangiitis (EGPA), known as Churg-Strauss syndrome, and microscopic polyangiitis (MPA). Other diseases associated with ANCA include drug-induced vasculitis and vasculitis limited to the kidneys [1]. The disease most commonly involves the upper and lower respiratory tracts and the kidneys, often presenting as a pulmonary-renal syndrome, and lung involvement is frequent and associated with increased mortality. Pulmonary manifestations are ambiguous and require differentiated treatment approaches [2,3].

The pathogenesis of GPA is complex and has many factors in its development. The main cellular pathology pathway is associated with cytoplasmic ANCA (c-ANCA) directed against proteinase 3 (PR3), which is present in 75-90% of cases [1]. Genetic predisposition, environmental factors (particularly infections such as persistent carriage of *Staphylococcus aureus*), and immune dysregulation, including activation of neutrophil and formation of inflammatory granules in the vessels, seem to be involved in the development of this reaction [3].

Typically, microscopic angiomatosis, which is a proliferation of small vessels within inflamed tissue, may present with nonspecific systemic symptoms, fever, joint pain, muscle pain, and weight loss. Other symptoms that may occur include urinary symptoms, cough with or without hemoptysis, skin symptoms, and other nonspecific symptoms from other systems. Without appropriate treatment, these symptoms may progress and even lead to the patient's death [4]. Recently, the treatment has evolved. Initially, patients achieve remission with glucocorticoids and cyclophosphamide or rituximab, where azathioprine or maintenance doses of rituximab are then used to maintain it [5,6]. One of the new therapies that is used in the application is avacopan (C5a receptor inhibitor), which is the main factor influencing the efficacy in reducing glucocorticoids, as well as its side effects [6]. Although therapeutic options are available for clinical complications, this disease is characterized by recurrence, and immunosuppressive therapy often causes significant toxicity. Early diagnosis and prompt treatment are crucial for favorable outcomes.

## Case presentation

A 57-year-old man was transferred to the nephrology department from another hospital for evaluation and management of acute renal failure. Since December 2019, he had experienced low-grade fever, malaise, and abdominal and lower back pain. He denied hematuria and night sweats but reported occasional urinary urgency. The referring unit documented progressive renal failure (creatinine rising from 6.9 mg/dL to 11 mg/dL; urea 210 mg/dL (Table 1). Urinalysis showed proteinuria, hematuria, and pyuria, with urine output decreasing to 500 mL/day (Table 2). Inflammatory markers were elevated: C‑reactive protein 130 mg/L and procalcitonin 1.23 ng/mL (Table 1). His medical history included hypertension and chronic right-eye inflammation (uveitis treated with dexamethasone drops, diagnosed by an ophthalmologist and under his supervision). There was a family history of cancer in both parents. He denied smoking and alcohol use.

**Table 1:** Laboratory findings ANCA, antineutrophil cytoplasmic antibody.

Date	Parameter				
	Creatinine (mg/dL)	Urea (mg/dL)	CRP (mg/L)	Procalcitonin (ng/mL)	Anti-ANCA antibodies
8.02.2020	6.9	210	130	1.23	-
13.02.2020	11.41	232.4	158.97	0.913	-
15.02.2020	9.78	172.4	-	-	-
17.02.2020	7.88	116.6	139.3	-	-
18.02.2020	-	-	-	-	Positive
19.02.2020	7.43	109.3	-	-	-
21.02.2020	8.69	-	167.71	-	-
27.02.2020	-	145.2	11.32	-	-
01.03.2020	8.17	-	24.32	-	-
06.03.2020	-	-	24.18	-	-
11.03.2020	-	-	-	1.3	-
13.03.2020	-	-	121.71	2.04	-
15.03.2020	8.5	-	-	-	-
16.03.2020	-	-	35.47	1.62	-
19.03.2020	6.01	-	16.43	-	-
21.03.2020	7.6	64.3	11.5	-	-
Reference range	0.7-1.2	16.6-48.5	<5	<0.1-0.5 µg/L	Negative

**Table 2:** Urinalysis performed on 13.02.2020

Parameter	Result	Reference range
Color	Pale yellow	-
Clarity	Clear	-
pH	5	4.8-7.4
Specific gravity	1.01 g/mL	1.005-1.03 g/mL
Glucose	Negative	Negative
Ketones	Negative	Negative
Protein	0.88 g/L	Negative
Bilirubin	Negative	Negative
Urobilinogen	Negative	Negative
Nitrite	Negative	Negative
Bacteria	150/µL	Negative
White blood cells	45/µL	0-20/µL
Red blood cells	1116/µL	0-7/µL
Yeasts	Negative	Negative
Crystals	Negative	Negative
Epithelial cells	Negative	Negative
Mucus	Negative	Negative
Pathologic casts	Positive	Negative
Hyaline casts	Negative	0-5

**Figure 1:**
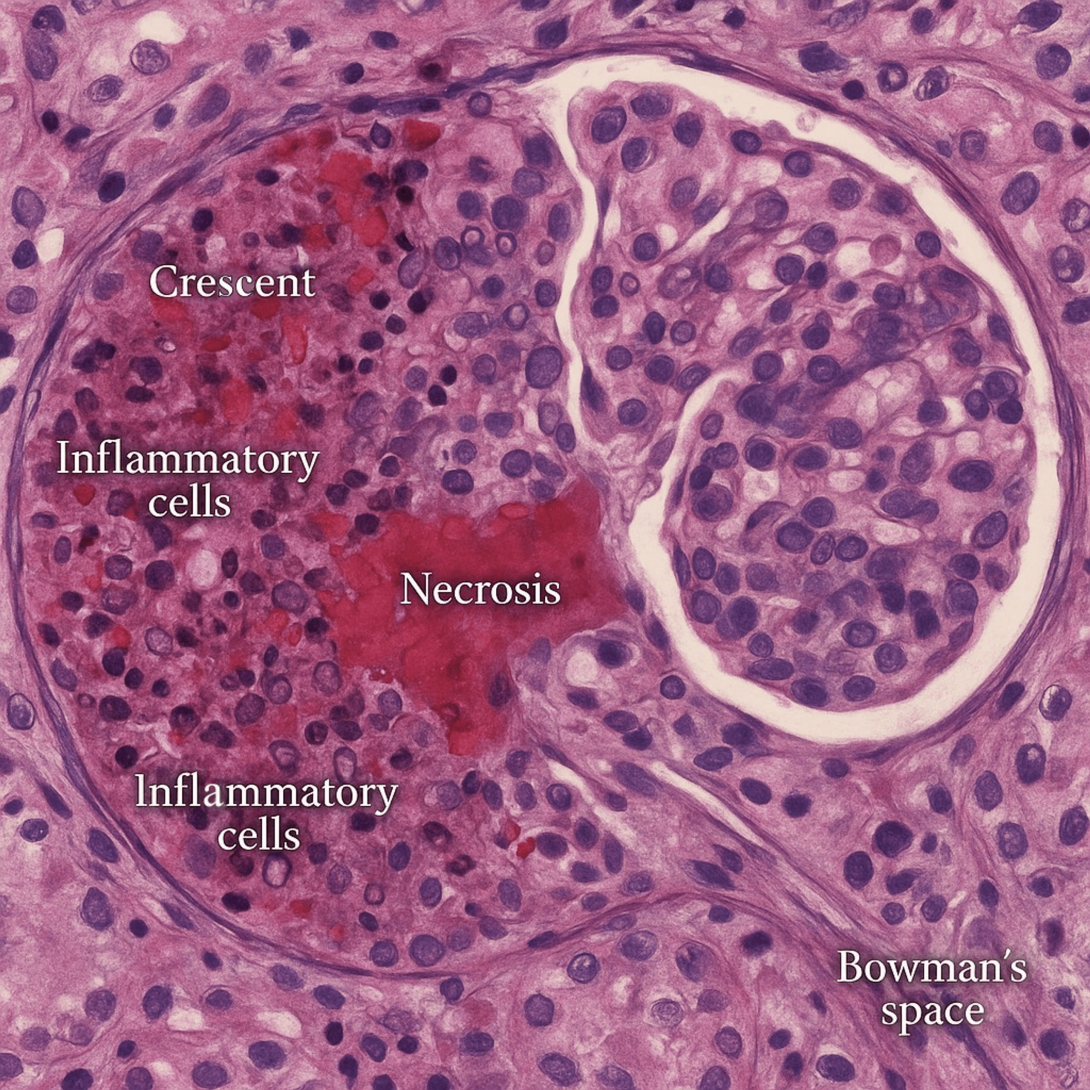
Histological section demonstrating necrotizing pauci-immune glomerulonephritis with cellular crescents Magnification 400x, hematoxylin and eosin staining.

**Figure 2:**
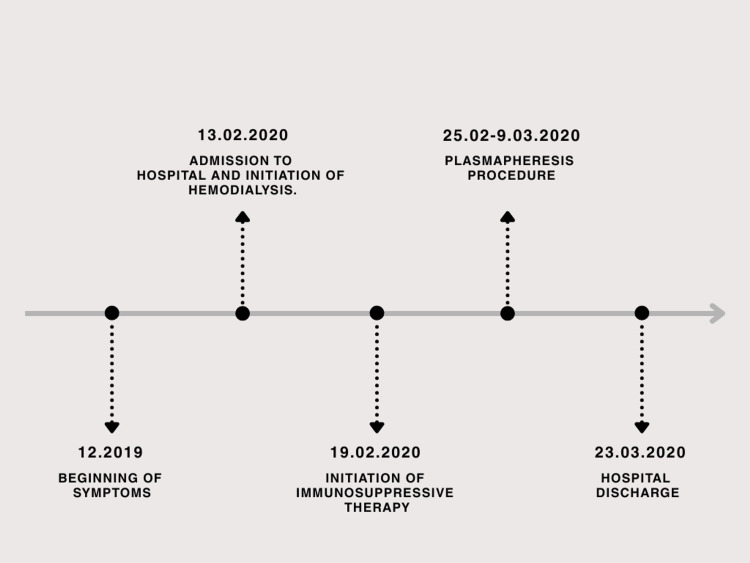
Timeline of clinical course

On admission on 13.02.2020, a right internal jugular central venous catheter was placed, and hemodialysis was initiated. Cefuroxime (1.5 g every 8 h) therapy was continued. During the first week, he developed self-limited purpura on the lower limbs and became anuric. Due to the lack of improvement after antibiotic therapy (8 days) and based on the patient’s clinical presentation, a suspicion of systemic vasculitis was raised. On February 19, a kidney biopsy was performed and showed pauci-immune necrotizing glomerulonephritis with cellular crescents (Table 3, Figure 1). Serologic testing was positive for PR3‑ANCA. Immunosuppressive therapy was started with three pulses of methylprednisolone IV (500 mg each), followed by oral methylprednisolone (16 mg) 48 mg daily and oral cyclophosphamide (2 mg/kg) 150 mg daily. On 24.02.2020, a non-tunneled central venous catheter was replaced with a tunneled catheter via the left internal jugular vein; in the right jugular vein, thrombosis was observed, where the non-tunneled catheter was placed, which is a common complication. From February 25 to March 9, the patient underwent seven sessions of plasma exchange. After treatment, clinical improvement was observed, including the return of urine output (400-700 mL/day) and a reduction in inflammatory markers. On March 11, he developed fever, hypotension, coffee‑ground vomiting, and worsening inflammatory markers. Broad-spectrum antibiotics (ceftazidime and vancomycin) were started, and immunosuppressive therapy was held. Endoscopy and imaging revealed no new pathology. Cyclophosphamide was permanently discontinued due to bone marrow suppression. Methylprednisolone was resumed at 16 mg/day. The patient’s clinical and laboratory condition gradually improved. He was discharged home in stable condition. The timeline of the patient’s clinical course is presented in Figure 2.

**Table 3:** Preliminary assessment of the morphology of the kidney biopsy

Category	Description
Material	Sections include fragments of the renal cortex and medulla with a total length (after embedding) of approximately 1.7 cm.
Glomeruli (20)	Cellular crescents are visible in 19 glomeruli, mostly global. Segmental necrosis of the vascular loop in 8 glomeruli. Segmental sclerosis of the vascular loop in 3 glomeruli. Collapse of the vascular loop in parts of segments adjacent to crescents. Segmental lysis of Bowman’s capsule in some glomeruli. Segmental fibrous thickening of Bowman’s capsule in some glomeruli.
Interstitial	Extensive inflammatory infiltrate of mononuclear cells in the stroma without stromal fibrosis
Tubules	Tubulitis swelling of the epithelial cytoplasm in some tubules. Exfoliation and flattening of the epithelium in some tubules. No tubular atrophy.
Arterial vessels (cross-sections of interlobular caliber arteries, arterioles)	Moderately developed arteriolar hyalinosis
Staining for the presence of amyloid deposits was negative.	
IFL examination revealed the presence of	Fibrinogen deposits within the crescents.
IFL examination did not reveal the presence of	IgA, IgG, IgM, C1q, C3, or λ and κ light chain deposits in the examined kidney.
Electron microscopic evaluation	Performed on sections through 2 glomeruli. The width of the capillary loops was variable - some loops with narrowed lumina due to collapse of their walls. The basement membranes of some capillary loops were thin, approximately 250 nm in thickness, with segmental folding. In many capillary loops, mesangiolysis was observed. Podocytes were reduced in number, their foot processes flattened along most of the basement membranes, with preservation over small segments.
Diagnosis	Necrotizing pauci-immune glomerulonephritis with cellular crescents in the majority of glomeruli available for evaluation. Acute tubular epithelial injury. Moderately developed arteriolar hyalinosis.

## Discussion

The clinical case described above presents a severe form of renal GPA, associated with vasculitis mediated by ANCA antibodies (AAV). During hospitalization, the patient required urgent renal hemodialysis therapy and immunosuppressive therapy. A renal biopsy confirmed progressive, necrotizing pauci‑immune glomerulonephritis, a pattern commonly linked to PR3‑ANCA. Despite the timely initiation of methylprednisolone pulses, cyclophosphamide, and plasma exchange, the disease course was complicated by infection and hematologic toxicity, necessitating treatment modifications.

Current guidelines endorse glucocorticoids combined with either cyclophosphamide or rituximab as standard induction therapy for organ‑threatening GPA, although both approaches carry substantial risks, including cytopenia and infection, as observed in this case [7]. Studies such as the RAVE and RITUXVAS trials have established rituximab as a non-inferior alternative to cyclophosphamide, particularly in relapsing cases [7]. Long‑term observational data indicate ongoing infection risk with rituximab, with serious infections reported at rates near 7-8 per 100 patient‑years in maintenance cohorts, underscoring the need for vigilant monitoring [8]. Given the clinical context, relapse risk appears high and should be incorporated into ongoing management decisions [9].

In the current therapy, it is important to achieve the right balance between the efficacy and toxicity of treatment. In the latest scientific studies, it has been shown that new drugs, such as avocopan (C5a receptor antagonist) and tecilizumab (IL-6 receptor blocker), have the potential to reduce the use of glucocorticoids, which causes adverse effects [7]. In this case, cyclophosphamide was discontinued due to bone marrow suppression, emphasizing the need to individualize therapy based on toxicity and response. For example, rituximab could help with adverse effects. During remission monitoring, biomarkers such as C‑reactive protein (CRP) and urinary MCP‑1 can aid assessment, alongside clinical evaluation and renal parameters [10].

This case underscores the importance of prompt diagnosis supported by histopathology and ANCA serology, as well as vigilant monitoring for treatment-related complications. It also reflects a common dilemma in GPA management: how to control severe disease without incurring prohibitive toxicity, especially in patients with renal involvement.

## Conclusions

In summary, this case illustrates the severe and complex renal presentation of GPA, highlighting the importance of early diagnosis, timely initiation of appropriate treatment, vigilant monitoring, and prevention of adverse effects. Access to newer therapies such as avacopan and rituximab may reduce steroid‑related toxicity and improve quality of life. This case also underscores the need to develop better treatment strategies while maintaining individualized therapy as a core principle. Ultimately, a multidisciplinary approach and long‑term follow‑up are essential to optimize outcomes and minimize the risk of relapse or treatment‑related harm.
